# Toxoplasmosis: A Timeless Challenge for Pregnancy

**DOI:** 10.3390/tropicalmed8010063

**Published:** 2023-01-13

**Authors:** Tuba Damar Çakırca, İlkay Nur Can, Melis Deniz, Ayşe Torun, Çiğdem Akçabay, Ahmet Güzelçiçek

**Affiliations:** 1 Department of Infectious Diseases and Clinical Microbiology, Sanliurfa Training and Research Hospital, Sanliurfa 63250, Turkey; 2 Department of Pediatric Infectious Diseases, Sanliurfa Training and Research Hospital, Sanliurfa 63250, Turkey; 3 Department of Obstetrics and Gynecology, Division of Maternal Fetal Medicine, Sanliurfa Training and Research Hospital, Sanliurfa 63250, Turkey; 4 Department of Pediatrics, Harran University Faculty of Medicine, Sanliurfa 63290, Turkey

**Keywords:** congenital toxoplasmosis, congenital infections, pregnancy, prenatal diagnosis, spiramycin prophylaxis

## Abstract

This study aimed to evaluate the prevalence of toxoplasmosis in pregnant women, as well as the general characteristics, clinical and laboratory findings, and pregnancy and fetal outcomes of pregnant women diagnosed with acute toxoplasma infection (ATI). The toxoplasma IgM, IgG, and IgG avidity test results of pregnant women who applied to our referral hospital between January 2016 and June 2022, and among them, those diagnosed with ATI, were analyzed. The 119 patients diagnosed with ATI during this time period were included for further analysis. The prevalence of toxoplasmosis in pregnant women was found to be 46.2%, and the rate of ATI was 4%. The total mother-to-child transmission rate was 5% (5/101). Congenital toxoplasmosis (CT) was observed in 1 (1.1%) child of the 87 pregnant women who received spiramycin prophylaxis, though it was found in 4 (30.8%) of the children of the 13 untreated mothers. With respect to prenatal treatment, CT rates were significantly higher in the children born to untreated mothers (*p* = 0.001). In conclusion, although toxoplasma seroprevalence was found to be high in our region, there was a paucity in diagnosis, follow-up, and treatment. Our findings support that prenatal spiramycin prophylaxis is effective in preventing the transmission of parasites from mother to child.

## 1. Introduction

Toxoplasmosis is a parasitic infection which can be seen both in humans and animals. Its clinical presentation is primarily asymptomatic or mild in immunocompetent individuals. However, it may cause serious problems in immunosuppressed patients and in the infants of mothers who had such an infection during pregnancy. Congenital toxoplasmosis (CT), which develops in children who contract the infection vertically from their mothers, affects the lives of many individuals [1]. The severity of fetal or of a newborn’s disease depends on the gestational age at which the mother was infected, the parasitic load, the virulence of the strain, and the mother’s immune system [2]. Clinical manifestations range from mild symptoms to serious consequences such as retinochoroiditis, hydrocephalus, microcephaly, mental retardation, and even death. Some cases may show no evidence of infection at birth but develop late clinical symptoms, resulting in neurological and ocular sequelae [3]. Further, an infection may cause undesirable outcomes of pregnancy, such as miscarriage, premature birth, intrauterine growth retardation (IUGR), and stillbirth [1].

Considering that this infection is typically asymptomatic, it is highly important to perform screening tests during pregnancy to detect possible cases [2,4]. A diagnosis of acute toxoplasma infection (ATI) in pregnant women can be made with serological tests of toxoplasma IgM, IgG, and IgG avidity. Screening tests for toxoplasmosis are not mandatory in Turkey, but the Ministry of Health encourages family physicians to examine every pregnant woman in their first trimester. On the other hand, such a preventable and treatable disease that has so many detrimental consequences for newborns is an issue that deserves more attention.

In this study, we aimed to evaluate the prevalence of toxoplasmosis in pregnant women, as well as the general characteristics, clinical and laboratory findings, and pregnancy and fetal outcomes of pregnant women diagnosed with ATI in our referral hospital in Şanlıurfa Province, Turkey.

## 2. Materials and Methods

This retrospective and single-center study was conducted at the Şanlıurfa Training and Research Hospital, which is a tertiary referral center that provides services in obstetrics and gynecology. Pregnant women admitted to our referral hospital between January 2016 and June 2022 were evaluated for toxoplasmosis, and those diagnosed with ATI were included for further analysis.

### 2.1. Patient Selection

**Figure 1:**
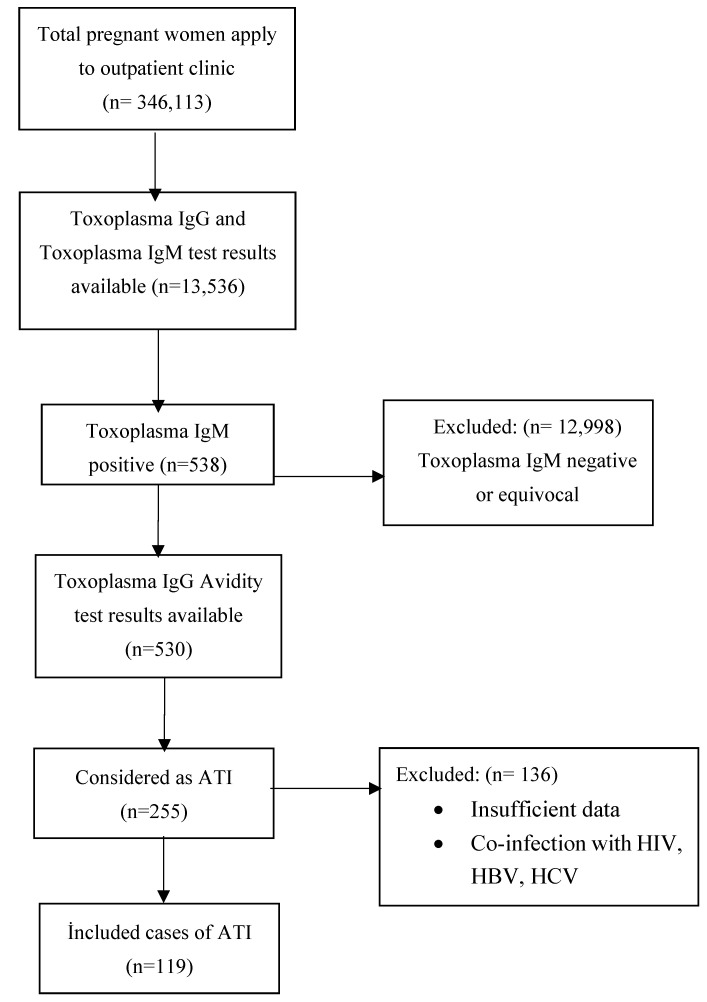
Flow chart of the patient selection.

Between January 2016 and June 2022, 346,113 pregnant women were admitted to the Şanlıurfa Training and Research Hospital. Of these, 21,177 underwent analysis for toxoplasma IgG and toxoplasma IgM, but only 13,536 test results were available. In 538 of the patients, the toxoplasma IgM result was found to be positive. An avidity test was requested for 530 of these patients. After the evaluation of the avidity test results, 255 women were considered to have ATI. Patients who were co-infected with HIV, HBV, and HCV; those who were administered immunosuppressive therapy; and those whose complete data could not be accessed were excluded from the study. Finally, 119 patients were included in the study (Figure 1).

A diagnosis of ATI is made as follows [1]:

1. Patients who have a positive toxoplasma IgM and low IgG avidity results within each trimester;
2. Patients who have a positive toxoplasma IgM and high or intermediate IgG avidity results after 16 weeks of pregnancy (in which acute past distinction cannot be made).

The newborns of all patients diagnosed with ATI during pregnancy were evaluated for CT. A diagnosis of CT is made as follows [5,6]:

1. Those who have toxoplasma IgM test positivity in the first 10 days after birth;
2. Detection of increasing or persistent toxoplasma IgG titer in the first year of life without treatment;
3. Detection of T. gondii DNA PCR or a positive toxoplasma IgM test for cerebrospinal fluid (CSF);
4. Sabin-Feldman test positivity in blood or CSF (a titer of 1:16 is considered to be positive).

The demographic features, clinical characteristics, laboratory findings, administered treatments, and outcomes of the patients and their newborns were evaluated using the hospital’s record system. Patients were also contacted by phone for missing data, and they were asked about neonatal outcomes, mode of delivery, and whether the pregnancy was ongoing.

### 2.2. Laboratory Analysis

The toxoplasma IgG and IgM assays were performed by conventional laboratory methods on a Cobas 8000 analyzer (Roche, Penzberg, Germany), and the toxoplasma IgG avidity results were obtained with a chemiluminescent microparticle immunoassay on an Abbott Architect. Toxoplasma DNA was detected using polymerase chain reactions in BioRad CFX 96 real-time PCR. The Sabin-Feldman Dye Test (SFDT) was carried out with live antigen and methylene blue staining in accordance with the technique of the Parasitology Reference Laboratory (Refik Saydam Hygiene Center, Ankara, Turkey).

### 2.3. Statistical analyses

Statistical analyses were completed using SPSS version 21.0 (SPSS Inc., Chicago, IL, USA). Data are presented as medians (minimum–maximum) and as n (%).

### 2.4. Ethical Permission

This study was approved by the Harran University School of Medicine Ethics Committee Commission, with the protocol number HRU/22.13.01.

## 3. Results

In this study, the toxoplasma IgG was positive in 6252 (46.2%) of 13,536 pregnant patients included in the analysis, while the toxoplasma IgM test was found to be positive in 538 (4%). The maternal characteristics of the patients are shown in Table 1.

The median (minimum–maximum) patient age was 26 years (18–40). The median week of pregnancy at diagnosis was 12 (5–38). During manuscript submission, 112 patients had given birth and 7 pregnancies were ongoing. Of the 112 pregnancies, 2 of them were ended with abortion. One of the women who had miscarriage was a 25-weeks-pregnant woman whose amniocentesis toxoplasma PCR result was positive. After diagnosis, 3 patients decided to terminate their pregnancies voluntarily. Of the 107 pregnant women, 6 gave birth prematurely.

Avidity test results were low in 98 (82.4%), grayzone in 8 (6.7%), and high in 13 (10.9%) patients at the time of diagnosis. After the diagnoses were made, amniocentesis was recommended to all patients whose gestational week was appropriate. Only 24 patients accepted the procedure, and in one patient, the amniocentesis toxoplasma PCR found to be positive and their pregnancy was ended with abortion. The spiramycin prophylaxis was started after the diagnosis was made. A total of 16 patients rejected treatment. The median duration of treatment was 17 (2–33) weeks for the remaining patients.

The status and outcome data for 18 children was not available, whereas no anomalies were detected in 96 children and a diagnosis of CT was made in 5 children. The mother of one child had a positive PCR test result from the amniotic fluid and terminated her pregnancy by means of abortion. The total mother-to-child transmission rate was 5% (5/101). Clinical, laboratory, and maternal features of the children diagnosed with CT after birth are shown in Table 2.

Child-1 was firstly suspected to have CT during the fetal USG, which showed macrocephaly and hydrocephalus. After birth, chorioretinite was also found during the ocular examination. The mother of Child-2 was diagnosed with ATI at birth, and she was not diagnosed and treated during pregnancy. The child had a toxoplasma scar, visual defects, and strabismus during the ocular examination, as well as cerebellar hypoplasia and an arachnoid cyst, which was found during magnetic resonance imaging (MRI). Child-3 was diagnosed with CT while seeking the etiology of its microcephaly. The mother had been diagnosed with ATI one week before birth, but she had not received any treatment. Child-4 had been suspected of having a congenital infection after heavy IUGR findings on a fetal USG. After the tests on the mother were found to be positive for ATI, the child was born prematurely and was transferred to another hospital. When the family was contacted to determine the child’s outcome, it was learned that he had died.

CT was observed in 1 (1.1%) child of the 87 pregnant women who received spiramycin treatment, though it was observed in four (30.8%) of the children of the 13 untreated mothers. When the rates of congenital spread to the infants of patients who received or did not receive spiramycin were compared, there was a statistically significant difference between the two groups (*p* = 0.001). The mother whose child was infected with T. gondii, despite using treatment, was diagnosed with ATI when she was 20 weeks pregnant. The avidity test result was low, which means she had definitely received the infection during pregnancy, and a positive PCR test result from the amniotic fluid confirmed the diagnosis. Treatment was started immediately, but she miscarried after five weeks of spiramycin prophylaxis and before we received the PCR test result from the amniotic fluid.

## 4. Discussion

In this retrospective single-center study, the prevalence of toxoplasmosis in pregnant women was found to be 46.2% and the rate of ATI was 4%. It was also observed that 5 of the 101 mothers diagnosed with ATI transmitted the infection to their children, and the total transmission rate was found to be 5%. When the mothers who received treatment and those who did not receive treatment were compared, the CT rates were significantly higher in the children of those who did not receive treatment.

Toxoplasmosis is one of the most common parasitic infections threatening public health in the world, and serological evidence of the infection is detected in nearly one third of the global population. [7]. The prevalence rates vary from region to region, and they reach as high as 90% in some parts of Africa and up to 60% in some parts of Europe [7,8,9]. However, data from Asia (including China and India) are limited on this topic [10]. Due to the geographical region and climatic conditions of our country, which is suitable for easy parasitic spread, the prevalence of toxoplasmosis in pregnant women has been reported to be 18.3% and 94.6% in different regions of Turkey over the last twenty years [11]. In a recent study from the west of Turkey, the prevalence of toxoplasmosis in pregnant women was found to be 27.78% [12]. Another previous study from the southeast of Turkey, which was conducted in the same province as our study, reported a high prevalence of toxoplasmosis (69.5%) [13]. In our region, social habits and food culture, such as the high consumption of raw meat, may be the reason for the high prevalence, and in this study, we also reported a high prevalence (46.2%) in pregnant women. Recognizing the regional prevalence of toxoplasmosis may be the first starting point for taking precautions with pregnant women to prevent the mother-to-child transmission of such a devastating disease.

Although the risk of transmission from mother to child in early pregnancy is low, the risk of anomaly in the child is high. On the contrary, as the gestational weeks progress, the rate of transmission increases and the rate of damage to the child decreases [1,10,14]. Transmission rates in pregnancy are globally reported to be 29% [15]. Donadona et al. reported that the transmission rate in women who seroconvert during pregnancy was 32.9%, but it was 4.7% in women who were suspected to have toxoplasmosis during pregnancy [16]. In another study, evidence of infection was found in the fetuses of 4.3% of 346 women [17]. Hotop et al. also reported a low transmission rate of 4.8% in pregnant women receiving treatment [18]. Due to a lack of follow-up, only patients diagnosed with the infection during pregnancy were included, but the cases who developed seroconversion during pregnancy were not included in our study, and the transmission rate from mother to child was found to be 5%. Our findings are consistent with the literature, but they may be low for our high-prevalence region. It is likely that many cases were missed because of difficulties in diagnosis, and it is likely that not all pregnant women in our region, where the fertility rate is high, were screened.

There are different approaches in different countries regarding toxoplasma screening programs during pregnancy. Pregnant women are screened three times during pregnancy in Austria and monthly in France. In the USA (in Massachusetts and New Hampshire), a newborn screening program for congenital toxoplasmosis is in place for all newborns. In Brazil, where prevalence is high, a perinatal screening program for pregnant women and newborns was launched in early 2022. However, Spain and Switzerland do not carry out any screening programs [19]. Indeed, regular prenatal examination during pregnancy has been demonstrated to have the benefit of reducing the risk of vertical transmission [20]. Although there is no mandatory screening program in our country, family physicians are encouraged by the Ministry of Health to examine every pregnant woman in their first trimester. The fact that children are still encountering the various morbidities of CT suggests that screening programs would be beneficial for such a preventable infection.

The morbidity of CT, which is one of the most severe clinical entities caused by T. gondii, is very high in humans. In a study conducted in France, where most of the information about CT was obtained, 11 of the 272 fetuses with CT were lost before birth. The outcome was unknown for 27 infants, and 206 of 234 live-born infants were asymptomatic, while 21 had moderate and 7 had severe infections [21]. In another study carried out in Austria, where the prevalence is high, clinical manifestations were observed in 10.6% of the studied children (15/141) and all of them had chorioretinitis, while 12 had cerebral findings [22]. Although the triad of hydrocephalus, chorioretinitis, and cerebral calcification directly indicates CT, the most common presentation of the disease is ocular involvement (strabismus, microphthalmy, cataracts, and even blindness), in which late-onset detriment may occur [10]. Wallon et al. detected retinal damage in 24% of children with CT within six years of follow-up [23]. In our study, two of four children had both ocular and brain involvement, whereas one child had only microcephalus. However, one of the children who was born with severe IUGR could not be evaluated because he was lost in the early postnatal period. A proven intrauterine fetal infection can be determined by positive toxoplasma gondii PCR from amniotic fluid [2]. In a study conducted in Norway, 4% (14/346) of the studied women who underwent amniocentesis had a positive result for toxoplasmosis [17]. In another study by Prusa et al., 34 of 707 (4.8%) patients who underwent amniocentesis were found to be positive [22]. Similarly, in our study, PCR was positive in 4.2% (1/24) of the patients who accepted amniocentesis; unfortunately, the child was lost due to abortion. We do not know the outcome of ongoing pregnancies or the status of other aborted and voluntarily terminated fetuses.

The management of toxoplasmosis in pregnancy varies from country to country, though spyramicine is preferred in most centers. In some centers, the combination of pyrimethamine and sulfadiazine is chosen for treating confirmed fetal infections or for the late-pregnancy period. Additionally, in some countries, spyramicine therapy is provided first, and then the pyrimethamine and sulfadiazine combination treatment administered on an ongoing basis [18]. However, there are countries where the pyrimethamine and sulfadiazine combination is not available, and a dual therapy of spyramicine and trimethoprim–sulfamethoxazole is used [24]. In Turkey, spyramicine is provided to every pregnant woman diagnosed with ATI, independent of the trimester and whether amniocentesis is performed, and when a PCR test for T. gondii is found to be positive, the treatment is changed to pyrimethamine, sulfadiazine, and folinic acid. On the other hand, spyramicine treatment is ongoing until delivery if a PCR test result is negative or if amniocentesis is not performed. In studies investigating the efficacy of treatment, it was reported that parasite transmission from the mother to the child was higher in untreated pregnant women, and spyramicine prophylaxis was found to be effective for preventing CT. In addition, it has been shown that early diagnosis and treatment can reduce the mother-to-child transmission of the parasite, and can also reduce the disease’s severity in the infected fetus [18,20,25,26]. However, the SYROCOT study did not find robust evidence for a link between early treatment and decreased risk of CT [14]. Additionally, in a European cohort, prenatal treatment was not shown to reduce the risk of retinochoroiditis [27]. There are studies comparing the performance of treatment regimens, as well. Buonsenso et al. investigated the effectiveness of spyramicine and trimethoprim–sulfamethoxazole treatment in the transmission of toxoplasma from mother to child, and they found that the spyramicine and trimethoprim–sulfamethoxazole therapy was superior to the spyramicine monotherapy. This combination was non-inferior to a pyrimethamine–sulfadiazine combination therapy [24]. In our study, 103 of the patients were given a spiramycin monotherapy, whereas 16 patients refused to take medicine. Considering the patients whose pregnancies ended, as is consistent with the literature, we observed that the rate of congenital spread to infants was significantly higher in patients who did not receive spiramycin than in those who had received such treatment. The authors concluded that spiramycin may be effective in preventing mother-to-child transmission of the parasite.

This study has some limitations. First, because of the retrospective design, the patient data were insufficient, which limited our ability to evaluate all patients effectively. After giving birth, some of the patients did not bring their children to the hospital for examination, and so not all children could be consulted for a pediatric infectious disease, which may have resulted in potential cases of CT being overlooked. Because of the high missed-follow-up rate, the mother-to-baby infection transmission rates may have been low. In addition, a definite prenatal diagnosis could not be made because most of the pregnant women refused to undergo amniocentesis, and so the treatment of the mothers with spiramycin prophylaxis had to be continued, which is why we could not present our experience with the pyrimethamine, sulfadiazine, and folinic acid combination therapy.

## 5. Conclusions

In this study, we presented a single-center prevalence of toxoplasmosis in pregnant women, which was found to be 46.2%, while the rate of ATI was 4% and the mother-to-child transmission rate was 5%. Our findings support that prenatal spiramycin prophylaxis is effective in preventing the transmission of parasites from mother to child. However, it was concluded that although toxoplasma seroprevalence was found to be high in our region, there was a paucity in diagnosis, follow-up, and treatment. The first starting point would be to identify the regional prevalence of toxoplasmosis in order to take precautions for pregnant women. In essence, to prevent CT, for which mortality and morbidity is high, early diagnosis and treatment of pregnant women with ATI is necessary. Regular screening tests would be beneficial for this purpose in high-prevalence areas.

## Figures and Tables

**Table 1:** Maternal characteristics and outcomes of acute toxoplasma infection in pregnant women.

***Age, years, median (min-max)***	**26 (18–40)**
***Gestational age at presentation***	
≤12 weeks (first trimester), n (%)	60 (50.4)
13–27 weeks (second trimester), n (%)	46 (38.7)
≥28 weeks (third trimester), n (%)	13 (10.9)
***Gravity, median (min-max)***	3 (1–10)
***Parity, median (min-max)***	1 (0–8)
***Fetal USG examination***	
Normal, n (%)	114 (95.8)
Abnormal, n (%)	5 (4.2)
***Type of delivery***	
Normal vaginal, n (%)	71 (59.6)
Cesarean section, n (%)	36 (30.3)
Voluntarily termination, n (%)	3 (2.5)
Missed abortion, n (%)	2 (1.7)
Ongoing pregnancy, n (%)	7 (5.9)
***Week of pregnancy at diagnosis, median (min-max)***	12 (5–38)
***Week of pregnancy at delivery, median (min-max)***	38 (17–40)
***Toxoplasma IgM, median (min-max)***	2.06 (1.01–27.56)
***Toxoplasma IgG, median (min-max)***	293 (2–650)
***Toxoplasma IgG avidity***	
Low avidity (<50%), n (%)	98 (82.4)
Intermediate avidity (50–50.9%), n (%)	8 (6.7)
High avidity (≥60%), n (%)	13 (10.9)
***Toxoplasma PCR in amniotic fluid***	
Not done, n (%)	95 (79.8)
Done—PCR negative, n (%)	23 (19.3)
Done—PCR positive, n (%)	1 (0.8)
***Treatment***	
Spiramycin, n (%)	103 (86.6)
Rejected treatment, n (%)	16 (13.4)
***Treatment duration, week, median (min-max)***	17 (2–33)
***Newborn abnormalities***	
Absent, n (%)	96 (80.7)
Present, n (%)	5 (4.2)
Unknown, n (%)	18 (15.1)

PCR: polymerase chain reaction. Data are presented as median (minimum–maximum) and n (%).

**Table 2:** Clinical, laboratory and maternal features of children diagnosed with congenital toxoplasmosis.

	CHİLD-1	CHİLD-2	CHİLD-3	CHİLD-4
***Maternal Characteristics***				
**Week of Pregnancy at Diagnosis**	37	38	37	29
**Week of Pregnancy at Delivery**	39	38	38	29
**Intrauterine** **Amniocentesis**	Not Done	Not Done	Not Done	Not Done
**Intrauterine Treatment**	No	No	No	No
**Type of Delivery**	C/S	Vaginal	Vaginal	C/S
**Toxoplasma IgM**	Negative	Positive	Positive	Positive
**Toxoplasma IgG**	Positive	Positive	Positive	Positive
**Toxoplasma IgG Avidity**	Low	Low	No Data	Low
***Fetal Characteristics***				
**Gender**	Female	Male	Male	Male
**Fetal USG Findings**	Macrocephaly Hydrocephalus	Not Done	Not Done	Heavy IUGR
**Birth Weight**	2800	2950	2500	650
**Apgar Score**	6/8	9/10	No Data	No Data
**Toxoplasma IgM**	Negative	Positive	Positive	Not Adjusted
**Toxoplasma IgG**	Positive	Positive	Negative	Not Adjusted
**Sabin-Feldman**	1/1024 (Blood)	1/1024 (Blood)	1/64 (CSF)	Not Adjusted
**BOS Toxo PCR**	Negative	No Data	Negative	Not Adjusted
**Clinical Findings of Toxoplasmosis**	Chorioretinite Hydrocephalıs	Toxoplasma scar Cerebellar hypoplasia Arachnoid cyst	Microcephaly	Not Adjusted
**Treatment**	Primetamine Sulfadiazine Leucoverine IV Steroid	Primetamine Leucoverine Clindamycin	Primetamine Sulfadiazine Leucoverine	No Treatment
**Outcome**	Alive	Alive	Alive	Ex

C/S: Cesarean section, CSF: cerebrospinal fluid.

## Data Availability

The data presented in this study are available in this article and on request from the corresponding author. The data are not publicly available to ensure the privacy of the study participants.
